# *Viola betonicifolia*-Mediated Biosynthesis of Silver Nanoparticles for Improved Biomedical Applications

**DOI:** 10.3389/fmicb.2022.891144

**Published:** 2022-05-20

**Authors:** Yingping Jang, Xiaoya Zhang, Rongxue Zhu, Songlin Li, Shiyu Sun, Wenqiang Li, Hao Liu

**Affiliations:** ^1^Department of Rehabilitation Medicine, Guangdong Second Provincial General Hospital, Guangzhou, China; ^2^Engineering Technology Research Center for Sports Assistive Devices of Guangdong, Guangzhou Sport University, Guangzhou, China; ^3^Department of Chinese Medicine, Guangdong Second Provincial General Hospital, Guangzhou, China

**Keywords:** biosynthesis, silver NAPs, antibacterial, antifungal, biofilm inhibition

## Abstract

We report the biosynthesis of silver (Ag) nanoparticles (NAPs) (LEVB-Ag NAPs) by an environmentally friendly green synthesis approach using the phytoconstituents of *Viola betonicifolia* leaf extract. The spectroscopic techniques were employed to characterize biosynthesized LEVB-Ag NAPs successfully. Biosynthesized LEVB-Ag NAPs were assessed for antibacterial and antimycotic activities against bacterium and mycological strains (*H. pylori, S. epidermidis, C. tropicalis, and T. rubrum*) using the serial dilution method. They were also evaluated for their biofilm inhibiting potential against both bacterial and fungi species. They were further assessed for the cytobiocompatible potential with two normal cell lines (293T and hMSC). The results demonstrate that the biosynthesized LEVB-Ag NAPs showed superior log_10_ reduction in bacterial and fungal growth and presented more than 99.50% killing efficiency. Moreover, biosynthesized LEVB-Ag NAPs excellently inhibited the biofilm formation of bacterial (Gram-positive and Gram-negative) and mycological strains and presented more than 80% biofilm inhibiting percentage compared to both plant extract and CHE-Ag NAPs. They further presented good cytobiocompatibility *in vitro* with 293T and hMSC cells compared to CHE-Ag NAPs. Biosynthesized LEVB-Ag NAPs presented superior antibacterial, antimycotic, biofilm inhibition, and cytobiocompatible results that might be attributed to the synergistic effect of the NAPs’ physiochemical properties and the immobilized phytoconstituents from plant leaf extract on their surface. Hence, biosynthesized LEVB-Ag NAPs may be a promising contender for a variety of therapeutic applications.

## Introduction

The development of multi-drug resistance (MDR) in different bacterial strains (*Enterococci*, *Staphylococci*, *Klebsiella*, *Acinetobacter*, *Pseudomonas*, and *Enterobacter* species) has become a serious threat over the globe. Numerous antimicrobial agents have been developed (Cephalosporins, Carbapenems, Vancomycin, and Methicillin); however, with the rise of MDR in bacteriological species, the therapeutic efficacy of currently available antibiotics is at risk (Boucher et al., 2009; Khan et al., 2020). Additionally, certain fungi species, are also developing resistance to a variety of antifungal agents, such as azole (Berman and Krysan, 2020). Several antimicrobials have been developed; unfortunately, due to the rise of MDR in bacteria and fungi, the therapeutic efficacy of currently available agents is at risk. Microbial species demonstrate resistance to antimicrobials by enzymatic deactivation and alteration of drug target sites, as well as by lowering antibiotic cell wall permeability and exhibiting efflux mechanisms (Li and Nikaido, 2012; Khan et al., 2020). According to the (World Health Organization [WHO], 2022), bacterial infections now cause 0.7 million lives each year. If we do not discover effective treatments to manage or eliminate these dangerous bacteria and fungi, this figure might climb to ten million by 2050 (New report calls for urgent action to avert antimicrobial resistance crisis). Therefore, this has become obligatory to find alternate routes to tackle these MDR pathogenic microbes and cancers.

Nanotechnology, a promising and revolutionary field, has garnered much interest for its potential to address these issues. Nanotechnology is an interdisciplinary field of study that leverages fundamental concepts from many areas such as biology, physics, engineering and chemistry, and to create unique approaches for manipulating minute particles, culminating in the fabrication of nanoparticles (NAPs) (Khan and Lee, 2020a). Ag NAPs are particularly significant among them owing to their unique visual, physical, and chemical characteristics (Korbekandi et al., 2009; Thakkar et al., 2010; Majdalawieh et al., 2014). They are widely used as antitumor, anti-inflammatory, and antimicrobial agents in catheter coatings, medical equipment disinfection, antimicrobial filters, dental hygiene, eye care, and topical ointments (Swamy et al., 2015; Singh et al., 2016). Ag NAPs have a long history dating back to ancient times and a bright future in biological and chemical sciences.

There is a growing need to develop environmentally safe ways for synthesizing nanoparticles without using hazardous chemicals. A great deal of attention has recently been focused on developing environmentally friendly strategies for the production of metallic nanoparticles. Enzymes, microorganisms, and medicinal plants are eco-friendly nanoparticle manufacturing methods (Korbekandi et al., 2009; Thakkar et al., 2010; Khan et al., 2021a). Plant-mediated synthesis (green synthesis) of metallic nanoparticles has piqued the interest of scientists in the last decade since it is a straightforward and time-saving procedure compared to other biological approaches, such as microbial cultures (Ahmed et al., 2016; Singh et al., 2016; Ijaz et al., 2017; Lu et al., 2021; Wang et al., 2021). The use of plant leaf extracts is a promising approach due to their ease of preparation, low cost, stability, and scalability in comparison to other biomolecules (Khan and Lee, 2020a; Khan et al., 2020).

Moreover, in the production of NAPs, leaf extracts from plants have been used as both reducing and capping agents (Khan and Lee, 2020a). Metal NAPs have been shown to be produced by phytochemical constituents of plants such as polyphenols, terpenoids, alkaloids, and flavonoids that promote metal ion reduction and the formation of metal nanoparticles (Elbagory et al., 2019; Gharehyakheh et al., 2020). Furthermore, it is believed that biogenic phytomolecules may enhance NAPs’ inherent antioxidant, antibacterial, and anticancer capabilities compared to chemically synthesized NAPs (Chandran et al., 2006; Kumar and Yadav, 2009; Muthukumar et al., 2016). Consequently, the use of plant leaf extracts in green synthesis enhances the synergetic effect and increases the biocompatibility of nanoparticles (Khan and Lee, 2020a).

In this work, silver ions were bioconverted to NAPs using a *Viola betonicifolia* (L.) leaf extract, a *Violaceaceae* species. *V. betonicifolia* (L.) grows wild in Australia, Malaysia, Pakistan, Nepal, India, China, Sri Lanka, and Burma (Rizwan et al., 2019). As an antipyretic, astringent, purgative, anticancer, and diaphoretic, this plant has been used to treat a variety of maladies, including bronchitis, neurological disorders, cough, epilepsy, skin disorders, sinusitis, blood disorders, pharyngitis, pneumonia, and renal diseases (Iqbal and Hamayun, 2005; Rizwan et al., 2019). The extract of the entire leaves of *V. betonicifolia* has been found in several studies to have a high concentration of biogenic phytomolecules, including flavonoids, alkaloids, tannins, saponins, phenolic compounds, and triterpenoids (Muhammad et al., 2012, 2013a). Around 200 natural active compounds have been discovered and isolated from various *Viola* species (Zhu et al., 2015). Numerous plants have been employed in the green synthesis of Ag NAPs in the past; however, *V. betonicifolia* is not one of them (L.) as per the author’s best knowledge. Consequently, we disclose the biosynthesis of Ag NAPs using *V. betonicifolia* leaves extract for the first time. The antibacterial, anticancer, and antioxidant characteristics of biologically produced Ag NAPs were examined in light of the health benefits of *V. betonicifolia* leaf extract in the biomedical field.

## Materials and Methods

All chemicals [AgNO_3_ (209139), PBS (P3813), DMSO (C6164), tryptic soy broth (B311-150-S), Sabouraud-gentamicin-chloramphenicol (89579), RPMI agar (R8758), and phenazine methosulfate (P9625)] employed in this study were acquired from Merck (Darmstadt, Germany) and Sigma Chemicals Co (St. Louis, MS, United States). Mueller-Hinton broth (CM0405B), CellROX™ Green (C10444), Hoechst 33342 and PI staining kit (V13244) were purchased from Thermo Scientific™. Dulbecco’s Modified Eagle’s Medium (51435C), cell proliferation kit I (MTT) (11465007001), and cell proliferation kit II (XTT) (11465015001) were acquired from Merck (Darmstadt, Germany) and Sigma Chemicals Co (St. Louis, MS, United States). For the comparative biological study, commercially available chemically synthesized Ag NAPs (795925) were purchased from Sigma-Aldrich and denoted as CHE-Ag NAPs.

### *V. betonicifolia* Leaf Extract Preparation

**FIGURE 1:**
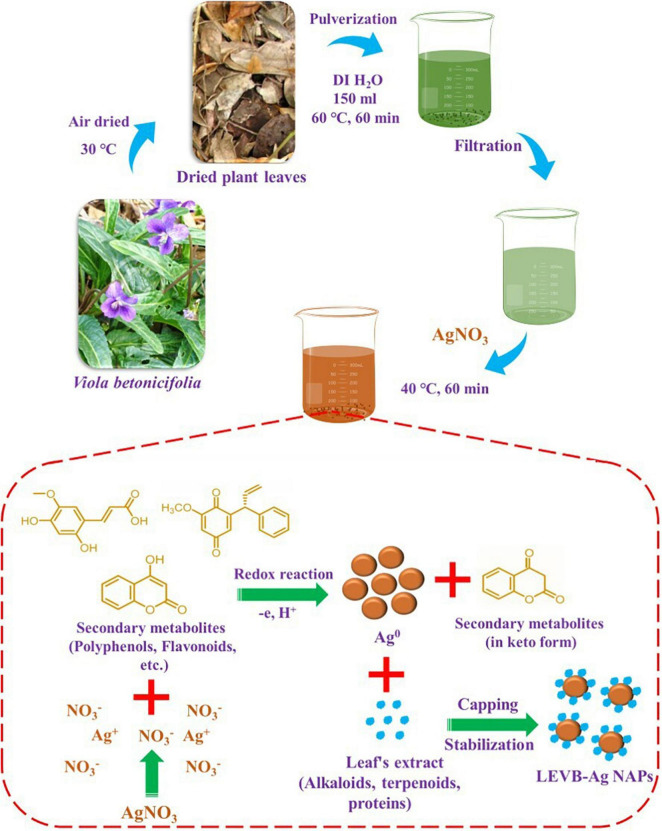
Schematic presentation for the plant-mediated biosynthesis and synthesis mechanism of LEVB-Ag NAPs using the leaf extract of *Viola betonicifolia*.

The fresh *V. betonicifolia* leaves were collected from the surrounding areas of Lahore, Pakistan. Its identification was made by Dr. Zaheer (Department of Botany, GC University, Pakistan). 20 g of fresh *V. betonicifolia* leaves were used to make the *V. betonicifolia* leaves extract. To eliminate any pollutants or dust, the leaves were carefully washed with deionized (DI) water and air-dried at 30°C. The dried leaves were chopped into tiny pieces and crushed in a grinder before being transferred to a 500 mL beaker. The 150 mL DI water was then added and heated for 60 min at 60°C. After that, the *V. betonicifolia* leaves extract was cooled to room temperature. Leave extract was then filtered and kept in an airtight glass container at 4°C for subsequent use (Figure 1).

### Biosynthesis of Silver Nanoparticles (LEVB-Ag NAPs)

One mM of AgNO_3_ was mixed in 25 mL of *V. betonicifolia* leaves extract for the green synthesis of silver nanoparticles (Figure 1). The resultant mixture was heated at 40°C for 60 min while stirring constantly. The formation of silver nanoparticles was identified when the reaction solution’s color changed from yellow to dark brown. The silver nanoparticles were removed from the reaction mixture by centrifugation at 3,000 rpm for 30 min. After centrifugation, the resulting silver nanoparticles were washed three times with DI/ethanol, dried at 40°C, and then calcined for 3 h in a muffle furnace at 200°C. Then these biosynthesized silver nanoparticles were named as LEVB-Ag NAPs and were used for further characterization.

### Characterization

Powder X-ray diffraction (XRD) spectroscopy was used to evaluate the crystalline nature and phase purity of the LEVB-Ag NAPs, which was performed in a Bruker D_2_ PHASER with LYNXEYE XE-T detector (Haidian, Beijing, China) with a wavelength (λ) of 0.154 nm. With a scanning rate of 1°/min and a slit width of 6.0 mm, the XRD spectra were obtained in the 2θ range from 10°–90°. The chemical composition of produced LEVB-Ag NAPs was determined using an energy-dispersive X-ray (EDX) spectroscopy system (Thermo Fisher Scientific Ultradry (Madison, WI, United States) connected to a scanning electron microscope. TEM images of the LEVB-Ag NAPs were taken using a Tecnai F 12 microscope (FEI/Philips Tecnai 12 BioTWIN, Baltimore, MD, United States) working at 200 kV acceleration voltage. The samples were dissolved in methanol and sonicated at 25–30°C before being put onto a carbon-coated copper grid for TEM examination. After draining the excess solution, the copper grid was left to dry for 5–10 min. UV–Visible spectroscopy was used to evaluate the optical characteristics of the LEVB-Ag NAPs. The materials were first dissolved in DI water and subsequently sonicated for 5 min at 25–30°C before being used in the UV-Vis experiment. The spectra were recorded as a function of wavelength using a Shimadzu 1700 spectrophotometer (Shimadzu, Columbia, Maryland, United States) from 250 to 650 nm at 25–30°C.

### Antibacterial Propensity

Two bacterial species, namely, *Helicobacter pylori* (ATCC^®^43504™) and *Staphylococcus. epidermidis* (ATCC 12228) were used to test the antibacterial potential of the LEVB-Ag NAPs. Both bacteria were purchased from American Type Culture Collection (ATCC, Manassas, VA, United States) and stored at −80°C. The bacteria strains were seeded onto separate blood agar plates and grown for 24 h at 37°C (Khan et al., 2021c). Following the growth of many bacterial colonies on the plates, they were diluted with phosphate buffer saline (PBS). Their cell density was maintained at 1 × 10^7^ colony forming units (CFU) per milliliter (mL). Following that, 10 mL of Mueller-Hinton broth (MHB) and 10 μL of each bacterial culture were put to wells of a 24-well microtiter plate. Each bacteria had a final concentration of 1 × 10^5^ CFU/mL in each well. After that, 100 μL of each sample dispersion in DI H_2_O at a 300 μg/mL concentration was transferred to separate wells and incubated for 24 h at 37°C. After that, the bacterial species in the wells were counted using the serial dilution plate counting technique. The antibacterial propensity was calculated using the following formulas and presented in log_10_ decrease in bacterial growth and percent killing.


$${\text{Log}}_{10}\,\text{reduction}={\text{log}}_{10}\,\left({\text{CFU}}_{\text{BI}}\right)-{\text{log}}_{10}\,\left({\text{CFU}}_{\text{AI}}\right),$$

$$\%\mathrm{killing}=\left({\text{CFU}}_{\text{BI}}-{\text{CFU}}_{\text{AI}}\right)/{\text{CFU}}_{\text{BI}}\times 100.$$

The CFU of bacterial strains before and after 24 h of incubation with the treatment of sample solutions, respectively, are CFU_BI_ and CFU_AI_.

### Live/Dead Bacteria Staining Assay

A confocal laser scanning microscope (CLSM, FV-1200, Olympus, Tokyo, Japan) was used to validate the antibacterial activity of LEVB-Ag NAPs using a live and dead bacterial staining experiment. The test was carried out according to the techniques described in Choi et al. (2017), Khan et al. (2021b). To achieve the stationary phase (about 10^5^–10^6^ CFU/mL), each bacterium was grown in nutritional broth in an orbital shaker at 37°C for 24 h. Each bacterium strain was injected onto sterilized cover glass covered with poly-L-lysine in a 24-well plate after incubation, and bacterial cells adhered to the cover glass were cultured for 1 h. After discarding the suspended bacterial cells, each cover glass was gently washed three times with a saline solution.

After being treated with 100 μL dispersion of LEVB-Ag NAPs in DI H_2_O at a 300 μg/mL concentration, the cells were incubated for 24 h at 37°C. According to the manufacturer’s instructions, bacteria cells on the cover glass were stained with Hoechst 33342 and PI. The CLSM images were taken using fluorescence microscopy (361/497 nm and 535/617 nm excitation/emission wavelengths for Hoechst 33342 and PI, respectively). For the live/dead staining experiment, we only investigated LEVB-Ag NAPs since they showed good antibacterial capabilities in terms of log_10_ decrease in bacterial growth.

### Reactive Oxygen Species Analysis

The CellROX^®^ Green was used to investigate the cause of bacteria death due to intracellular ROS production, as previously described (Arakha et al., 2015). 10^5^ CFU/mL of bacteria (*H. pylori* and *S. epidermidis*) were treated with 100 μL dispersion of LEVB-Ag NAPs in DI H_2_O at a 300 μg/mL concentration and then incubated at 37°C for 24 h. The microbial cells were incubated for another 30 min at 37°C with CellROX^®^ Green (5 μM). After then, CLSM was utilized to capture pictures with absorption wavelengths of 485 nm and emission wavelengths of 520 nm. The findings of NAPs-treated cells were compared to those treated with 1 mM H_2_O_2_ (positive control) and untreated cells (negative control).

### Antifungal Activity

*Candida tropicalis* (NRRLY 12968) and *Trichophyton rubrum* (ATCC^®^28188) were used to test the antifungal activity of the LEVB-Ag NAPs and were purchased from American Type Culture Collection (ATCC, Manassas, VA, United States). The same antibacterial activity technique was used as previously described in the antibacterial section, except seeding was done on a Sabouraud-gentamicin-chloramphenicol (SGC) fungal agar plate. The incubation temperature was maintained at 30°C (Khan et al., 2021c). The antifungal activity was calculated using the following formulas and presented in log_10_ reduction in bacterial growth and percent killing.


$${\text{Log}}_{10}\,\text{reduction}={\text{log}}_{10}\,\left({\text{CFU}}_{\text{BI}}\right)-{\text{log}}_{10}\,\left({\text{CFU}}_{\text{AI}}\right),$$

$$\%\mathrm{killing}=\left({\text{CFU}}_{\text{BI}}-{\text{CFU}}_{\text{AI}}\right)/{\text{CFU}}_{\text{BI}}\times 100.$$

The CFU of mycological strains before and after 24 h of incubation with the treatment of sample solutions, respectively, are CFU*_*BI*_* and CFU*_*AI*_*.

### Effectiveness of Biofilm Inhibition

In contrast to CHE-Ag NAPs and *V. betonicifolia* leaves extract, the biofilm inhibition efficacy of the LEVB-Ag NAPs was tested against bacterial (*H. pylori* and *S. epidermidis*) and mycological species (*C. tropicalis* and *T. rubrum*) (Khan et al., 2020, 2021c). In brief, mycological and bacterial biofilms were developed at 1 × 10^7^ CFU/mL in a 96-well plate using TSB and RPMI agar, respectively. Both microbial species were cultured for 24 h at 30°C and 37°C, respectively. Each well was rinsed three times with PBS after the planktonic cells were removed. After that, 100 μL of each sample dispersion in DI H_2_O at 300 μg/mL was added to each well, and the well plate was incubated at 37°C for 24 h. After washing each well with PBS, the staining agents (10 μL phenazine methosulfate and 90 μL XTT) were applied. After that, the microtiter well plate was held at 37°C in the dark for 4 h. Finally, at a wavelength of 492 nm, optical density (OD) was measured, and biofilm inhibition effectiveness was computed as a percentage using the formula:


$$\mathrm{Biofilm}\mathrm{inhibition}\left(\%\right)=\left[{\text{OD}}_{\text{UT}}-{\text{OD}}_{\text{T}}/{\text{OD}}_{\text{UT}}\right]\times 100,$$

The optical densities of untreated and treated microbial species are represented by OD_UT_ and OD_T_, respectively.

### Cytobiocompatibility Analysis

The MTT technique was used to assess the cytobiocompatibility of the LEVB-Ag NAPs against hMSC cells in comparison to the CHE-Ag NAPs and leaves extract of *V. betonicifolia*. The hMSC cells were cultured in Dulbecco’s Modified Eagle’s Medium (DMEM) at 37°C in a humidified environment of 5% CO_2_ and 95% air. The hMSC cells were grown in 100 μL of DMEM in a 96-well plate for 24 h at 37°C to achieve cell confluency of up to 5 × 10^8^ cells/well. Then 100 μL dispersion of CHE-Ag NAPs, *V. betonicifolia* leaves extract, and LEVB-Ag NAPs in DI H_2_O at a 300 μg/mL concentration were added to each well containing grown hMSC cells. The plate was incubated for another 24 h at 37°C. The plate was then centrifuged to remove the supernatant before rinsing with phosphate buffer saline (PBS) solution. The plate was then placed in an incubator for 4 h at 37°C, during which time 15 μL of MTT labeling agent (0.5 mg/mL) was added to each well; the plate was then placed in an incubator for 4 h at 37°C, during which time 150 μL of DMSO was added to each well to solubilize the undissolved formazan crystals. Using a Varian Eclipse spectrophotometer, the absorption maxima of formazan product in each well were determined at 570 nm. The following formula was used to compute the percentage of cell viability:


$$\%\mathrm{Cell}\mathrm{viability}={\text{OD}}_{\text{sample}}/{\text{OD}}_{\text{control}}\times 100.$$

OD refers to optical density.

### Statistical Analysis

The biological assays were performed in triplicates, and results are presented in Mean ± SD. Two-Way ANOVA analysis was performed to check significance at *p* < 0.05.

## Results and Discussion

### Synthesis Mechanism and Characterization

*V. betonicifolia* has a variety of biologically active secondary metabolites (Muhammad et al., 2012, 2013b; Zhu et al., 2015; Rizwan et al., 2019) that may be utilized to synthesize biologically active nanoparticles. As a result, in the manufacture of bioactive silver nanoparticles, we employed the leaf extract of this plant as reducing agents and stabilizing agents. Figure 1 depicts a proposed approach for producing LEVB-Ag NAPs using plant leaf extract. Polyphenols and flavonoids included in the leaf extract may have a role in reducing Ag^+^ ions to metallic form during the redox process. At the same time, natural compounds found in the leaf extract, including alkaloids, terpenoids, and saponins, may easily cap and transform the produced Ag^0^ into silver nanoparticles (LEVB-Ag NAPs). Hussain et al. (2019) have described a similar synthesis mechanism.

**FIGURE 2:**
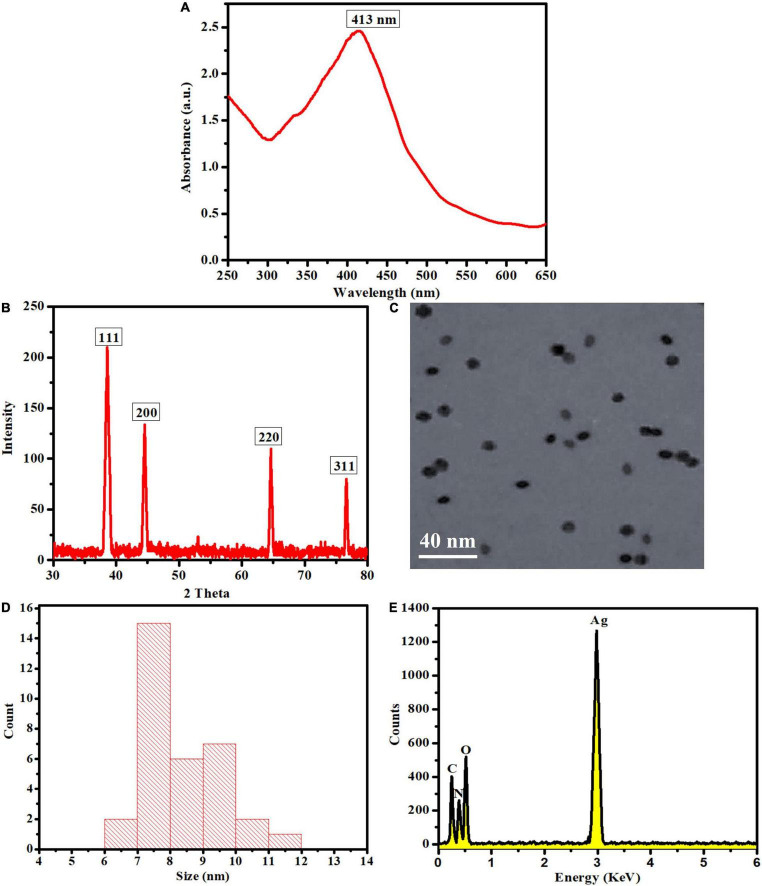
**(A)** UV-Visible spectra, **(B)** XRD, **(C)** TEM, **(D)** size distribution, and **(E)** EDX pattern of the biosynthesized LEVB-Ag NAPs.

A UV-Visible spectrophotometer was used to monitor the chemical reaction between the plant leaf extract and the dissolved silver ions. UV-Visible examination indicated that once the reaction was completed and the OE-Ag NPs were formed, an absorption peak at 413 nm was seen owing to the surface plasmon resonance phenomenon (Figure 2A; Alqahtani et al., 2020). Using XRD, the crystallinity and impurity of the biosynthesized LEVB-Ag NAPs were investigated. The peak’s intensity shows that the synthesized silver NAPs are highly crystalline. Additionally, the XRD peak positions are pretty similar to metallic silver’s, with no peaks associated with impure substances identified (Figure 2B). A similar XRD peaks pattern was also reported previously for the green synthesized silver NAPs with plant extract by Rajivgandhi et al. (2020a,b, 2022), Ma et al. (2021), Sellami et al. (2021). The morphology of the biosynthesized LEVB-Ag NAPs was determined using TEM. TEM images demonstrated that the LEVB-Ag NAPs have spherical morphology with uniform distribution with no agglomeration (Figure 2C). The particle size of the synthesized LEVB-Ag NAPs ranged from 6–11 nm, and the average particle size was 8.3 nm determined by TEM (Figure 2D). The elemental and compositional analysis was further carried out by using the EDX. The EDX spectrum displays that the sample comprises silver (Figure 2E). However, other peaks, such as C, and O, are also evident in the EDX spectrum due to the presence of plant phytomolecules on the surface of synthesized LEVB-Ag NAPs. Similar results of the EDX pattern were also reported by Kumar et al. (2019), Sellami et al. (2021) for the gold and silver nanoparticles synthesized by using leaf extract of different plants, respectively (Kumar et al., 2019; Sellami et al., 2021).

### Antibacterial Potential of LEVB-Ag NAPs

**FIGURE 3:**
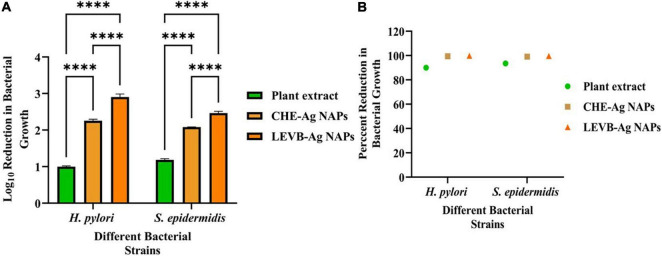
**(A)** Log10 reduction and **(B)** percent reduction in bacterial growth of Gram-positive and Gram-negative bacterial strains demonstrated by LEVB-Ag NAPs in comparison to plant leaf extract and CHE-Ag NAPs (****p < 0.0001).

Biosynthesized LEVB-Ag NAPs were investigated for antibacterial activities against Gram-positive and Gram-negative bacterial species in terms of Log_10_ reduction in bacterial growth. When comparing to a plant extract of leaves and CHE-Ag NAPs, the results revealed that biosynthesized LEVB-Ag NAPs had the most potent antibacterial activity and exhibited the highest Log_10_ and percent reduction in bacterial growth against all tested bacteriological strains (Figures 3A,B). Additionally, the extract of plant leaves revealed good antimicrobial activity against all microbiological strains tested. The improved bioactivity of LEVB-Ag NAPs against both bacteria might result from the synergistic impact of the NAPs’ physicochemical characteristics and immobilized bioactive components from the plant extract of leaves on their surface according to EDX investigations (Figure 2E).

**FIGURE 4:**
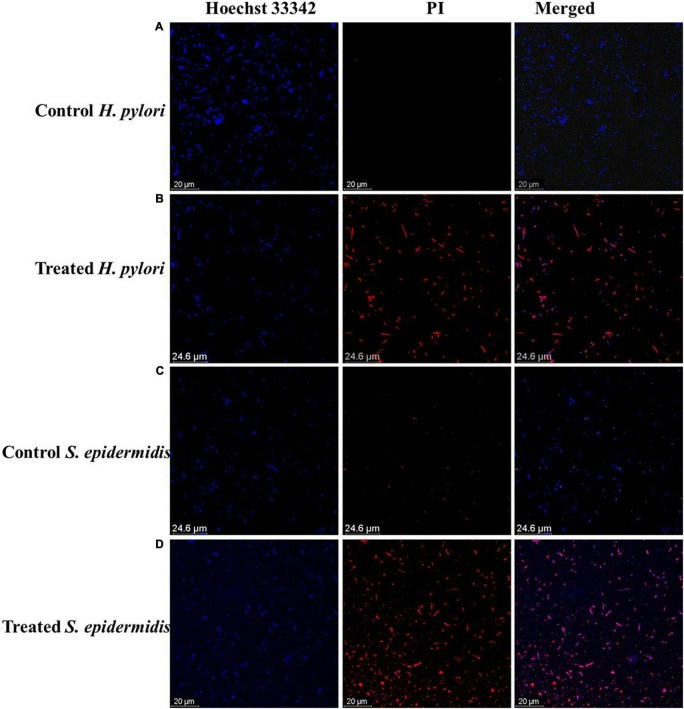
**(A,B)** control and treated *H. pylori*, respectively. **(C,D)** control and treated *S. epidermidis* respectively.

The antibacterial mode of action of the LEVB-Ag NAPs was then investigated using the live and dead staining assays. A live/dead staining experiment was performed utilizing a double staining kit (Hoechst 33342/PI) against two bacteria (*H. pylori* and *S. epidermidis*). These dyes are being used to distinguish between living and dead microbial cells. Hoechst 33342 is a persistent membrane dye that, when intercalated with DNA, may stain both living and dead cells. On the other hand, PI is an impermeant membrane dye that penetrates bacterium cells only when the cell membrane has been damaged. As a result, PI is employed to label dead cells (Ramalingam et al., 2016). As seen in Figures 4A,C, untreated bacterium strains stain exclusively with Hoechst 33342, suggesting that they are alive, and their cell membranes are undamaged. While bacteria treated with LEVB-Ag NAPs exhibited red fluorescence Figures 4B,D, indicating that their cell membrane had been damaged. These results imply that one mechanism driving LEVB-Ag NAPs’ antibacterial action may be the loss of bacterium membrane integrity.

**FIGURE 5:**
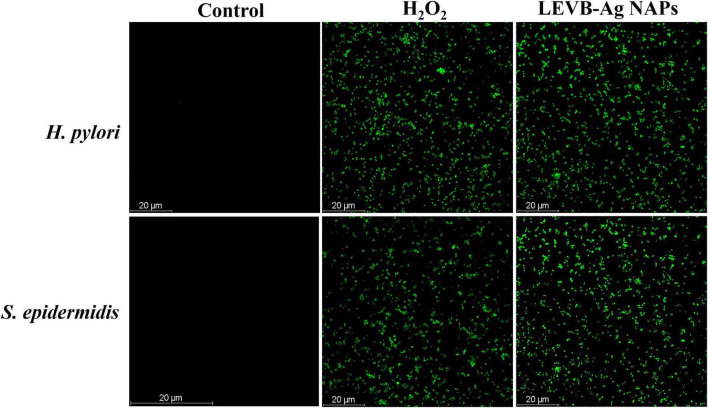
ROS generation in untreated (control), LEVB-Ag NAPs, and H_2_O_2_ treated Gram-positive and Gram-negative bacterial strains.

Microbial strains have been found to be destroyed by oxidative stress-mediated by intracellular ROS generation. Metal NAPs interact with a bacterium to form reactive oxygen species (ROS), which may cause oxidative stress and the death of organelles and biomolecules within the cell. The CellROX^®^Green assessment was conducted to detect oxidative stresses in bacterial cells after treatment with LEVB-Ag NAPs and H_2_O_2_, as shown in Figure 5. Under control, no intracellular ROS species were generated in any bacterial cell. The ROS generated by both bacteria treated with LEVB-Ag NAPs were equivalent to those produced by H_2_O_2_. These results show that the generation of ROS, which causes bacterium cells to die, might be one reason for the LEVB-Ag NAPs’ antimicrobial effect.

**FIGURE 6:**
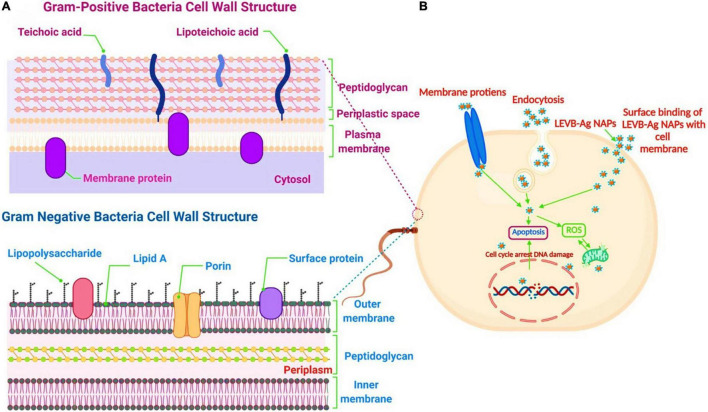
**(A)** The cell wall comparison between Gram-negative and Gram-positive bacteria. **(B)** Proposed antibacterial mechanism of LEVB-Ag NAPs against bacterial strains. Created with BioRender.com.

Biosynthesized LEVB-Ag NAPs were found to be more efficient in destroying Gram-negative bacteria versus Gram-positive bacteria. A possible explanation for the disparity is that NAPs are easier to penetrate inside Gram-negative bacteria and have a higher electrostatic affinity to surface-bound functionalities (such as sulfur proteins) than Gram-positive bacterium (Slavin et al., 2017; Yin et al., 2020). The ease with which biosynthesized LEVB-Ag NAPs penetrated could be explained because Gram-negative bacteria have quite a thinner cell wall than Gram-positive bacteria. Additionally, Gram-positive and Gram-negative bacteria have distinct cell wall structures and compositions (Pajerski et al., 2019), as seen in Figure 6A. Gram-positive bacteria possess a cell wall composed of multilayer channels of wall teichoic acid, lipoteichoic acid, and thick peptidoglycan. Both lipoteichoic acid and teichoic wall acid are further peptidoglycans- and cell membrane-associated. On the other hand, Gram-negative bacteria have a cell wall composed of outer/inner membrane layers, a thin coating of peptidoglycan, lipopolysaccharides, and a gel-like periplasm. Lipopolysaccharides are powerfully negatively charged macromolecules found only in Gram-negative bacterial strains’ outer membrane layers (Slavin et al., 2017; Pajerski et al., 2019; Yin et al., 2020). As a result, the biosynthesized LEVB-Ag NAPs have a more incredible inhibitory action on Gram-negative bacteria than Gram-positive bacteria.

We can propose a mechanism for bacterial cell death induced by LEVB-Ag NAPs after evaluating the rupture of cell membrane integrity and the production of intracellular oxidative stress caused by ROS species (Figure 6B). As a result of physical and oxidative disintegration, which led to the formation of numerous intracellular perturbations, LEVB-Ag NAPs demonstrated superior antibacterial activity. (1) The interactions of LEVB-Ag NAPs with cellular membrane proteins and other biomolecules such as lipopolysaccharides, etc., lead to cell membrane disintegration. (2) Cell membrane disintegration enhanced the membrane’s permeability to nanoparticle infiltration. (3) Membrane permeabilization may indeed cause physiological functionality spillage. (4) Upon penetrating, LEVB-Ag NAPs may bind with various biological compartments, generating oxidative stress and limiting their biological function even more. All of these aspects might contribute to the potential of LEVB-Ag NAPs to destroy Gram-positive and Gram-negative bacteria. A similar antibacterial mechanism was also reported by Sellami et al. (2021).

### Antimycotic Activity

**FIGURE 7:**
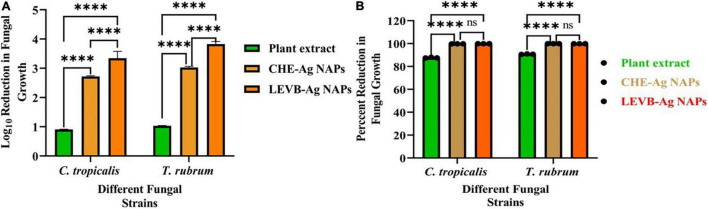
**(A)** Log10 reduction and **(B)** percent reduction in fungal growth demonstrated by LEVB-Ag NAPs in comparison to plant leaf extract and CHE-Ag NAPs (****p < 0.0001) (ns = *p* > 0.05).

In this study, the antimycotic activity of LEVB-Ag NAPs against *C. tropicalis* and *T. rubrum* was evaluated in terms of Log10 reduction in fungal growth. According to our findings, the superior antimycotic activity in terms of Log10 and percent reduction in fungal growth was demonstrated by biosynthesized LEVB-Ag NAPs than that of CHE-Ag NAPs and the plant leaf extract against all tested mycological strains (Figures 7A,B). Both plant leaf extract and CHE-Ag NAPs were also presented good antimycotic activity. It was interesting to note that the antimycotic activity of plant leaf extract against both mycological strains indicated the presence of biologically active phytoconstituents that can potentially destroy microbial strains (Muhammad et al., 2013c; Rizwan et al., 2019; Lu et al., 2021). Moreover, the higher antimycotic activity of LEVB-Ag NAPs compared to CHE-Ag NAPs might be due to the synergistic effect of the NAPs’ physicochemical properties and immobilized phytoconstituents from plant leaf extracts on their surface. Our current findings are in close agreement with previously reported studies (Muhammad et al., 2013c; Rizwan et al., 2019; Khan et al., 2020).

### Biofilm Inhibition Performance

**FIGURE 8:**
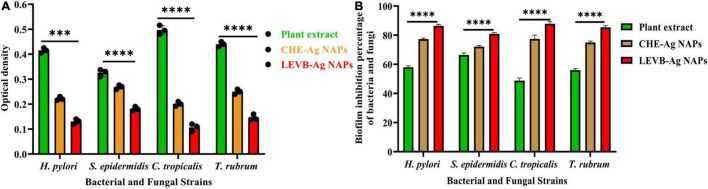
**(A)** the optical density and **(B)** biofilm inhibition performance of the biosynthesized LEVB-Ag NAPs against bacteriological and mycological strains compared to plant leaves extract, CHE-Ag NAPs (****p < 0.0001).

Numerous pathogens may survive in biofilms, which contributes significantly to their pathogenicity. Additionally, biofilms are the primary physiological structures involved in the development of bacterial resistance to a wide variety of medications. Numerous antibiotic and antifungal dosages are ineffective in killing bacteria and fungi embedded in biofilms (Khan and Lee, 2020b; Khan et al., 2021c). As a result, we investigated the biofilm inhibition effectiveness of biosynthesized LEVB-Ag NAPs against several microbial species. Figures 8A,B reveal that biosynthesized LEVB-Ag NAPs outperformed all other examined samples in terms of biofilm inhibiting performance against both bacterium and mycological species. Furthermore, plant leaf extract demonstrated good biofilm inhibiting performance against both strains of bacteria and fungi, which was ascribed to the inclusion of phytomolecules with biofilm inhibitory capabilities. The biofilm inhibition activity of CHE-Ag NAPs was found to be modest. The outstanding biofilm inhibiting efficiency of the LEVB-Ag NAPs might be attributed to an interplay between their physicochemical properties and the bound biologically active compounds from the leaf extract on their surfaces. Moreover, LEVB-Ag NAPs were shown to be more efficient in inhibiting bacterial biofilms of Gram-negative than Gram-positive. The results of biofilm inhibition performance are found in agreement with the antibacterial and antimycotic activities and the previous reported results (Rajivgandhi et al., 2019; Muthuchamy et al., 2020; Nadar Rajivgandhi et al., 2020; Govindan et al., 2022).

### Cytobiocompatibility Analysis of LEVB-Ag NAPs

**FIGURE 9:**
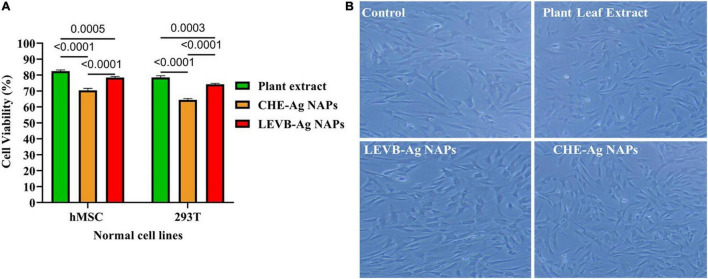
**(A)** Cytobiocompatibility of LEVB-Ag NAPs with hMSC and 293T cell lines in comparison to plant leaf extract and CHE-Ag NAPs. **(B)** Inverted microscope images of untreated (control) and treated hMSC cell lines with biosynthesized LEVB-Ag NAPs, plant leaf extract, and CHE-Ag NAPs.

Nanoparticles’ cytobiocompatibility with a variety of normal cells is crucial for their use in biological systems. Thus, we used the MTT assay to compare the cytobiocompatibility of biosynthesized LEVB-Ag NAPs to plant leaf extract and CHE-Ag NAPs in terms of cell viability% with normal cell lines (hMSC and 293T). When compared to other evaluated samples, the plant leaf extract was shown to be exceptionally biocompatible with both hMSC and 293T cell lines, demonstrating a high proportion of viable cells (Figure 9A). On the other hand, the biosynthesized LEVB-Ag NAPs displayed second higher cytocompatibility by exhibiting more percentage of viable cells of hMSC and 293T cell lines. Moreover, the CHE-Ag NAPs demonstrated least percentage of viable cells had the lowest cytocompatibility with hMSC and 293T cell lines. The findings also revealed that all examined materials seemed more cytobiocompatible with hMSC than the 293T cell lines.

Using an inverted microscope, we evaluated the morphological changes in hMSC cells treated with LEVB-Ag NAPs, plant extract, and CHE-Ag NAPs at a 120 μg/mL concentration. Figure 9B shows an inverted micrograph of hMSC cells. The images show that the morphology of hMSC cells remained similar to that of the untreated cells (control) after treatment with plant extract and LEVB-Ag NAPs. CHE-Ag NAPs, on the other hand, were slightly hazardous to hMSC cells, lowering their volume and cytoplasm while also changing their structure. Immobilization of bioactive and compatible biomolecules on the surface of LEVB-Ag NAPs may have contributed to their better cytobiocompatibility with normal cell lines. Another study found that immobilizing physiologically active phytoconstituents from plant extracts on the surface of silica NAPs improved their cytobiocompatibility (Rezaeian et al., 2021).

## Conclusion

We have successfully synthesized LEVB-Ag NAPs using phytoconstituents of *V. betonicifolia* leaf extract. Numerous spectroscopic techniques were employed to characterize biosynthesized LEVB-Ag NAPs successfully. The LEVB-Ag NAPs produced by biosynthesis showed superior antimicrobial activity against both microbiological species (bacteria and fungi). Additionally, they significantly reduced the production of biofilms by bacteria (Gram-positive and Gram-negative) and mycological strains. Biosynthesized LEVB-Ag NAPs seemed to be more biocompatible with 293T and hMSC cells than CHE-Ag NAPs. The superior biological capabilities of the LEVB-Ag NAPs might be a result of the synergistic effect of the immobilized phytoconstituents from plant leaf extract and the NAPs’ physiochemical properties.

As a consequence of this study biosynthesized LEVB-Ag NAPs may be a promising contender for a variety of biological and therapeutic applications. Further investigation is required to determine the dose-dependent biocompatibility *in vitro*/*in vivo*. Additionally, this study will open the way to produce biocompatible NAPs with enhanced biological functioning derived from medicinal vegetation.

## Data Availability Statement

The original contributions presented in the study are included in the article/supplementary material, further inquiries can be directed to the corresponding author/s.

## Author Contributions

YJ, XZ, RZ, and SL: conceptualization, methodology, software, validation, formal analysis, investigation, data curation, visualization, and writing—original draft preparation. SS, WL, and HL: resources and writing—review and editing. WL and HL: supervision, project administration, and funding acquisition. All authors have read and agreed to the published version of the manuscript.

## Conflict of Interest

The authors declare that the research was conducted in the absence of any commercial or financial relationships that could be construed as a potential conflict of interest.

## Publisher’s Note

All claims expressed in this article are solely those of the authors and do not necessarily represent those of their affiliated organizations, or those of the publisher, the editors and the reviewers. Any product that may be evaluated in this article, or claim that may be made by its manufacturer, is not guaranteed or endorsed by the publisher.

## References

[B1] Ahmed S. Ahmad M. Swami B. L. Ikram S. (2016). A review on plants extract mediated synthesis of silver nanoparticles for antimicrobial applications: A green expertise. *J. Adv. Res.* 7 17–28. 10.1016/j.jare.2015.02.007 26843966 PMC4703479

[B2] Alqahtani M. A. Al Othman M. R. Mohammed A. E. (2020). Bio fabrication of silver nanoparticles with antibacterial and cytotoxic abilities using lichens. *Sci. Rep.* 101:16781. 10.1038/s41598-020-73683-z 33033304 PMC7544908

[B3] Arakha M. Saleem M. Mallick B. C. Jha S. (2015). The effects of interfacial potential on antimicrobial propensity of ZnO nanoparticle. *Sci. Rep.* 5:9578. 10.1038/srep09578 25873247 PMC4397836

[B4] Berman J. Krysan D. J. (2020). Drug resistance and tolerance in fungi. *Nat. Rev. Microbiol.* 186 319–331. 10.1038/s41579-019-0322-2 32047294 PMC7231573

[B5] Boucher H. W. Talbot G. H. Bradley J. S. Edwards J. E. Gilbert D. Rice L. B. (2009). Bad bugs, no drugs: no ESKAPE! An update from the Infectious Diseases Society of America. *Clin. Infect. Dis.* 48 1–12. 10.1086/595011/2/48-1-1-TBL002.GIF 19035777

[B6] Chandran S. P. Chaudhary M. Pasricha R. Ahmad A. Sastry M. (2006). Synthesis of gold nanotriangles and silver nanoparticles using Aloe vera plant extract. *Biotechnol. Prog.* 22 577–583. 10.1021/bp0501423 16599579

[B7] Choi K.-H. H. Nam K. C. Lee S.-Y. Y. Cho G. Jung J.-S. S. Kim H.-J. J. (2017). Antioxidant Potential and Antibacterial Efficiency of Caffeic Acid-Functionalized ZnO Nanoparticles. *Nanomaterials* 7:148. 10.3390/nano7060148 28621707 PMC5485795

[B8] Elbagory A. M. Hussein A. A. Meyer M. (2019). The In Vitro Immunomodulatory Effects Of Gold Nanoparticles Synthesized From Hypoxis hemerocallidea Aqueous Extract And Hypoxoside On Macrophage And Natural Killer Cells. *Int. J. Nanomed.* 14 9007–9018. 10.2147/IJN.S216972 31819415 PMC6875510

[B9] Gharehyakheh S. Ahmeda A. Haddadi A. Jamshidi M. Nowrozi M. Zangeneh M. M. (2020). Effect of gold nanoparticles synthesized using the aqueous extract of Satureja hortensis leaf on enhancing the shelf life and removing *Escherichia coli* O157:H7 and Listeria monocytogenes in minced camel’s meat: the role of nanotechnology in the food indus. *Appl. Organomet. Chem.* 34:e5492.

[B10] Govindan R. Chackaravarthi G. Ramachandran G. Chelliah C. K. Muthuchamy M. Quero F. (2022). Effective removal of biofilm formation in *Acinetobacter baumannii* using chitosan nanoparticles loaded plant essential oils. *J. King Saud. Univ. Sci.* 34:101845. 10.1016/J.JKSUS.2022.101845

[B11] Hussain A. Alajmi M. F. Khan M. A. Pervez S. A. Hassan I. Khan R. A. (2019). Biosynthesized Silver Nanoparticle (AgNP) From Pandanus odorifer Leaf Extract Exhibits Anti-metastasis and Anti-biofilm Potentials. *Front. Microbiol.* 10:8. 10.3389/fmicb.2019.00008 30853939 PMC6396724

[B12] Ijaz F. Shahid S. Khan S. A. Ahmad W. Zaman S. (2017). Green synthesis of copper oxide nanoparticles using abutilon indicum leaf extract: antimicrobial, antioxidant and photocatalytic dye degradation activities. *Trop. J. Pharm. Res.* 16 743–753. 10.4314/tjpr.v16i4.2

[B13] Iqbal I. Hamayun M. (2005). Studies on the Traditional Uses of Plants of Malam Jabba Valley, District Swat, Pakistan. *Ethnobot. Leafl.* 2005:32.

[B14] Khan S. A. Lee C. S. (2020a). “Green Biological Synthesis of Nanoparticles and Their Biomedical Applications,” in *Applications of Nanotechnology for Green Synthesis. Nanotechnology in the Life Sciences*, eds Inamuddin Asiri A. (Cham: Springer), 247–280. 10.1007/978-3-030-44176-0_10

[B15] Khan S. A. Lee C. S. (2020b). Recent progress and strategies to develop antimicrobial contact lenses and lens cases for different types of microbial keratitis. *Acta Biomater.* 113 101–118. 10.1016/j.actbio.2020.06.039 32622052

[B16] Khan S. A. Shahid S. Ayaz A. Alkahtani J. Elshikh M. S. Riaz T. (2021a). Phytomolecules-coated NiO nanoparticles synthesis using abutilon indicum leaf extract: antioxidant, antibacterial, and anticancer activities. *Int. J. Nanomed.* 16 1757–1773. 10.2147/IJN.S294012 33688190 PMC7936927

[B17] Khan S. A. Shahid S. Hanif S. Almoallim H. S. Alharbi S. A. Sellami H. (2021b). Green Synthesis of Chromium Oxide Nanoparticles for Antibacterial, Antioxidant Anticancer, and Biocompatibility Activities. *Int. J. Mol. Sci.* 22:502. 10.3390/ijms22020502 33419098 PMC7825427

[B18] Khan S. A. Shahid S. Mahmood T. Lee C.-S. (2021c). Contact lenses coated with hybrid multifunctional ternary nanocoatings (Phytomolecule-coated ZnO nanoparticles:Gallic Acid:Tobramycin) for the treatment of bacterial and fungal keratitis. *Acta Biomater.* 128 262–276. 10.1016/j.actbio.2021.04.014 33866034

[B19] Khan S. A. Shahid S. Lee C.-S. S. (2020). Green Synthesis of Gold and Silver Nanoparticles Using Leaf Extract of Clerodendrum inerme; Characterization, Antimicrobial, and Antioxidant Activities. *Biomolecules* 10:835. 10.3390/biom10060835 32486004 PMC7356939

[B20] Korbekandi H. Iravani S. Abbasi S. (2009). Production of nanoparticles using organisms Production of nanoparticles using organisms. *Crit. Rev. Biotechnol.* 29 279–306. 10.3109/07388550903062462 19929319

[B21] Kumar I. Mondal M. Meyappan V. Sakthivel N. (2019). Green one-pot synthesis of gold nanoparticles using Sansevieria roxburghiana leaf extract for the catalytic degradation of toxic organic pollutants. *Mater. Res. Bull.* 117 18–27. 10.1016/J.MATERRESBULL.2019.04.029

[B22] Kumar V. Yadav S. K. (2009). Plant-mediated synthesis of silver and gold nanoparticles and their applications. *J. Chem. Technol. Biotechnol.* 84 151–157. 10.1002/jctb.2023

[B23] Li X. Z. Nikaido H. (2012). Efflux-Mediated Drug Resistance in Bacteria. *Drugs* 6912 1555–1623. 10.2165/11317030-000000000-00000 19678712 PMC2847397

[B24] Lu H. Zhang X. Khan S. A. Li W. Wan L. (2021). Biogenic Synthesis of MnO2 Nanoparticles With Leaf Extract of Viola betonicifolia for Enhanced Antioxidant, Antimicrobial, Cytotoxic, and Biocompatible Applications. *Front. Microbiol.* 12:761084. 10.3389/fmicb.2021.761084 34790185 PMC8591690

[B25] Ma D. Kanisha Chelliah C. Alharbi N. S. Kadaikunna S. Khaled J. M. Alanzi K. F. (2021). Chrysanthemum morifolium extract mediated Ag NPs improved the cytotoxicity effect in A549 lung cancer cells. *J. King Saud. Univ. Sci.* 33:101269. 10.1016/J.JKSUS.2020.101269

[B26] Majdalawieh A. Kanan M. C. El-Kadri O. Kanan S. M. (2014). Recent Advances in Gold and Silver Nanoparticles: synthesis and Applications. *J. Nanosci. Nanotechnol.* 14 4757–4780. 10.1166/JNN.2014.9526 24757945

[B27] Muhammad N. Rehman N. U. R. Khan H. Saeed M. Gilani A. H. (2013a). Prokinetic and laxative effects of the crude methanolic extract of Viola betonicifolia whole plant in rodents. *BMC Complement. Altern. Med.* 13:70. 10.1186/1472-6882-13-70 23530615 PMC3626539

[B28] Muhammad N. Saeed M. Khan H. Haq I. (2013b). Evaluation of n-hexane extract of Viola betonicifolia for its neuropharmacological properties. *J. Nat. Med.* 67 1–8. 10.1007/s11418-012-0636-0 22359189

[B29] Muhammad N. Saeed M. Qayum M. Khan H. (2013c). Antimicrobial screening of Viola betonicifolia. *Middle East J. Sci. Res.* 15 55–60. 10.5829/idosi.mejsr.2013.15.1.61254

[B30] Muhammad N. Saeed M. Khan H. (2012). Antipyretic, analgesic and anti-inflammatory activity of Viola betonicifolia whole plant. *BMC Complement. Altern. Med.* 12:59. 10.1186/1472-6882-12-59 22551220 PMC3419074

[B31] Muthuchamy M. Govindan R. Shine K. Thangasamy V. Alharbi N. S. Thillaichidambaram M. (2020). Anti-biofilm investigation of graphene/chitosan nanocomposites against biofilm producing P. aeruginosa and K. pneumoniae. *Carbohydr. Polym.* 230:115646. 10.1016/J.CARBPOL.2019.115646 31887894

[B32] Muthukumar T. Sudhakumari Sambandam B. Aravinthan A. Sastry T. P. Kim J. H. (2016). Green synthesis of gold nanoparticles and their enhanced synergistic antitumor activity using HepG2 and MCF7 cells and its antibacterial effects. *Process Biochem.* 51 384–391. 10.1016/j.procbio.2015.12.017

[B33] Nadar Rajivgandhi G. Ramachandran G. Chenthis Kanisha C. Li J. L. Yin L. Manoharan N. (2020). Anti-biofilm compound of 1, 4-diaza-2, 5-dioxo-3-isobutyl bicyclo[4.3.0]nonane from marine Nocardiopsis sp. DMS 2 (MH900226) against biofilm forming K. pneumoniae. *J. King Saud. Univ. Sci.* 32 3495–3502. 10.1016/J.JKSUS.2020.10.012

[B34] Pajerski W. Ochonska D. Brzychczy-Wloch M. Indyka P. Jarosz M. Golda-Cepa M. (2019). Attachment efficiency of gold nanoparticles by Gram-positive and Gram-negative bacterial strains governed by surface charges. *J. Nanoparticle Res.* 218:186. 10.1007/S11051-019-4617-Z

[B35] Rajivgandhi G. Maruthupandy M. Muneeswaran T. Anand M. Quero F. Manoharan N. (2019). Biosynthesized silver nanoparticles for inhibition of antibacterial resistance and biofilm formation of methicillin-resistant coagulase negative Staphylococci. *Bioorg. Chem.* 89:103008. 10.1016/J.BIOORG.2019.103008 31151056

[B36] Rajivgandhi G. N. Chackaravarthi G. Ramachandran G. Manoharan N. Ragunathan R. Siddiqi M. Z. (2022). Synthesis of silver nanoparticle (Ag NPs) using phytochemical rich medicinal plant *Lonicera japonica* for improve the cytotoxicity effect in cancer cells. *J. King Saud. Univ. Sci.* 34:101798. 10.1016/J.JKSUS.2021.101798

[B37] Rajivgandhi G. N. Maruthupandy M. Li J. L. Dong L. Alharbi N. S. Kadaikunnan S. (2020a). Photocatalytic reduction and anti-bacterial activity of biosynthesized silver nanoparticles against multi drug resistant Staphylococcus saprophyticus BDUMS 5 (MN310601). *Mater. Sci. Eng. C* 114:111024. 10.1016/J.MSEC.2020.111024 32994001

[B38] Rajivgandhi G. N. Ramachandran G. Maruthupandy M. Manoharan N. Alharbi N. S. Kadaikunnan S. (2020b). Anti-oxidant, anti-bacterial and anti-biofilm activity of biosynthesized silver nanoparticles using Gracilaria corticata against biofilm producing K. pneumoniae. *Colloids Surf. A Physicochem. Eng. Asp.* 600:124830. 10.1016/J.COLSURFA.2020.124830

[B39] Ramalingam B. Parandhaman T. Das S. K. (2016). Antibacterial Effects of Biosynthesized Silver Nanoparticles on Surface Ultrastructure and Nanomechanical Properties of Gram-Negative Bacteria viz. *Escherichia coli* and *Pseudomonas aeruginosa*. *ACS Appl. Mater. Interfaces* 8 4963–4976. 10.1021/acsami.6b00161 26829373

[B40] Rezaeian M. Afjoul H. Shamloo A. Maleki A. Afjoul N. (2021). Green synthesis of silica nanoparticles from olive residue and investigation of their anticancer potential. *Nanomedicine* 16 1581–1593. 10.2217/NNM-2021-0040 34169748

[B41] Rizwan K. Khan S. A. Ahmad I. Rasool N. Ibrahim M. Zubair M. (2019). A Comprehensive Review on Chemical and Pharmacological Potential of Viola betonicifolia: A plant with multiple benefits. *Molecules* 24:3138. 10.3390/molecules24173138 31470508 PMC6749243

[B42] Sellami H. Khan S. A. Ahmad I. Alarfaj A. A. Hirad A. H. Al-Sabri A. E. (2021). Green Synthesis of Silver Nanoparticles Using Olea europaea Leaf Extract for Their Enhanced Antibacterial, Antioxidant, Cytotoxic and Biocompatibility Applications. *Int. J. Mol. Sci.* 22:12562. 10.3390/IJMS222212562 34830442 PMC8621457

[B43] Singh P. Kim Y. J. Zhang D. Yang D. C. (2016). Biological Synthesis of Nanoparticles from Plants and Microorganisms. *Trends Biotechnol.* 34 588–599. 10.1016/j.tibtech.2016.02.006 26944794

[B44] Slavin Y. N. Asnis J. Häfeli U. O. Bach H. (2017). Metal nanoparticles: understanding the mechanisms behind antibacterial activity. *J. Nanobiotechnol.* 15:65. 10.1186/s12951-017-0308-z 28974225 PMC5627441

[B45] Swamy M. K. Akhtar M. S. Mohanty S. K. Sinniah U. R. (2015). Synthesis and characterization of silver nanoparticles using fruit extract of Momordica cymbalaria and assessment of their in vitro antimicrobial, antioxidant and cytotoxicity activities. *Spectrochim. Acta A Mol. Biomol. Spectrosc.* 151 939–944. 10.1016/j.saa.2015.07.009 26186612

[B46] Thakkar K. N. Mhatre S. S. Parikh R. Y. (2010). Biological synthesis of metallic nanoparticles. *Nanomed. Nanotechnol. Biol. Med.* 6 257–262. 10.1016/j.nano.2009.07.002 19616126

[B47] Wang M. Meng Y. Zhu H. Hu Y. Xu C. P. Chao X. (2021). Green synthesized gold nanoparticles using viola betonicifolia leaves extract: characterization, antimicrobial, antioxidant, and cytobiocompatible activities. *Int. J. Nanomed.* 16 7319–7337. 10.2147/IJN.S323524 34754187 PMC8570924

[B48] World Health Organization [WHO] (2022). *New Report Calls for Urgent Action to Avert Antimicrobial Resistance Crisis.* Available online at: https://www.who.int/news/item/29-04-2019-new-report-calls-for-urgent-action-to-avert-antimicrobial-resistance-crisis (accessed April 25, 2022).

[B49] Yin I. X. Zhang J. Zhao I. S. Mei M. L. Li Q. Chu C. H. (2020). The Antibacterial Mechanism of Silver Nanoparticles and Its Application in Dentistry. *Int. J. Nanomed.* 15:2555. 10.2147/IJN.S246764 32368040 PMC7174845

[B50] Zhu H. Qin S. S. Zhang N. Yang D. W. Han H. R. Wei K. H. (2015). Chemical Constituents and Biological Activities of Plants from the Genus Viola. *Chem. Biodivers.* 12 1777–1808. 10.1002/cbdv.201400240 26663836

